# Beliefs and attitudes about medications in patients with psychosis

**DOI:** 10.1192/j.eurpsy.2024.802

**Published:** 2024-08-27

**Authors:** K. Skaare-Fatland, M. Hermann, J. Assmus, M. Hartveit, T. Ruud, R. Horne, R. Drake, K. Drivenes, E. Biringer

**Affiliations:** ^1^Helse Fonna Health Trust, Haugesund; ^2^Western Norway University of Applied Sciences, Stord; ^3^ Helse Bergen Health Trust; ^4^University of Bergen, Bergen; ^5^University of Oslo, Oslo; ^6^Akershus University Hospital Trust, Lørenskog, Norway; ^7^UCL School of Pharmacy, University College London, London, United Kingdom; ^8^Vagelos College of Physicians and Surgeons, Columbia University, New York, United States; ^9^Directorate of e-health, Oslo, Norway

## Abstract

**Introduction:**

Patients’ beliefs and attitudes about medications play a role in whether they adhere to their medications or not. Knowledge on how beliefs and attitudes about medications can be influenced is therefore important.

**Objectives:**

The current study aimed to assess whether patients’ perceived support from their therapists regarding use of medications was associated with their beliefs and attitudes about medications. Because non-adherence in patients with psychosis frequently results in relapses and emergencies, this knowledge may be very useful for therapists and patients.

**Methods:**

This cross-sectional study included 310 patients diagnosed with psychosis from 31 clinical units in Norwegian mental health specialist care. We assessed beliefs about medications using the Beliefs about Medicines Questionnaire (BMQ). BMQ-specific consists of two subscales, BMQ-necessity and BMQ-concerns. Higher score on the necessity subscale indicates stronger beliefs in the necessity of taking the medicine. Higher score on the concern subscale indicates stronger concerns about taking the medicine. We used a newly developed self-report questionnaire, MedSupport, to assess the patients’ perceived support from therapists in dealing with their medications. Higher score on the MedSupport means that the patient experienced more support with decisions related to medications. Linear mixed effect models were used to investigate possible associations of sociodemographic factors, clinical factors and patients’ perceptions of medication support with BMQ.

**Results:**

Patients’ perceptions of medication support from therapists were positively associated with positive beliefs towards medications, β = 0.20, 95% CI [0.04 to 0.35], *p*=0.012, and negatively associated with concerns about taking the medications, β = -0.31, 95% CI [-0.44 to -0.17], *p* < 0.001, when other relevant variables were taken into consideration.

**Conclusions:**

The present study shows that therapists may affect patients’ beliefs and concerns about medications. Consequently, medication support may lead to improved adherence to medications prescribed.

**Disclosure of Interest:**

None Declared

